# Optimization of Low‐Contrast Detectability in Abdominal Imaging: A Comparative Analysis of PCCT, DECT, and SECT Systems

**DOI:** 10.1002/mp.17717

**Published:** 2025-03-03

**Authors:** Jessica D. Flores, Erik Wåhlin, Louise Blomkvist, Rebecca Titternes, Antonios Tzortzakakis, Bryan Connolly, Adrian Szum, Johan Lundberg, Patrik Nowik, Tobias Granberg, Gavin Poludniowski

**Affiliations:** ^1^ Department of Clinical Neuroscience Karolinska Institutet Stockholm Sweden; ^2^ Department of Nuclear Medicine and Medical Physics Karolinska University Hospital Stockholm Sweden; ^3^ Department of Clinical Science Intervention and Technology Karolinska Institutet Huddinge Sweden; ^4^ Department of Neuroradiology Karolinska University Hospital Stockholm Sweden; ^5^ Siemens Healthineers Solna Sweden

**Keywords:** model observers, multiple alternative forced choice, photon‐counting computed tomography

## Abstract

**Background:**

Clear representation of anatomy is essential in the assessment of pathology in computed tomography (CT). With the introduction of photon‐counting CT (PCCT) and more advanced iterative reconstruction (IR) algorithms into clinical practice, there is potential to improve low‐contrast detectability in CT protocols. As such, it is necessary to perform task‐based assessments to optimize protocols and compare image quality between PCCT and energy‐integrating CT, like dual‐energy CT (DECT) and single‐energy CT (SECT).

**Purpose:**

This work aimed to assess low‐contrast detectability in abdominal protocols used in clinical PCCT, DECT, and SECT, using both model and human observers.

**Methods:**

Data were acquired with the standard resolution scan mode on a PCCT (NAEOTOM Alpha, Siemens Healthineers, Forchheim, Germany) and a DECT/SECT (SOMATOM Force, Siemens Healthineers, Forchheim, Germany). Detectability was investigated in the CTP 515 low‐contrast module of the Catphan 600 phantom, which was surrounded by a fat annulus to simulate an abdomen and resulted in a water equivalent diameter of 298 mm. Supra‐slice contrast rods with a nominal 1.0% contrast and diameters of 4, 6, 9, and 15 mm were used. Factory abdominal protocols were adjusted to acquire images with various tube potentials (70, 90, 120, and 140 kV in PCCT; 70/150Sn and 80/150Sn kV in DECT; 100 and 120 kV in SECT), virtual monoenergetic image (VMI) energy levels (40 to 140 keV in PCCT and DECT), doses (5, 10 mGy in PCCT; 10 mGy in DECT and SECT), and IR settings (Br40 kernel, no quantum IR (QIR) and QIR levels 1 to 4 in PCCT; advanced modeled IR (ADMIRE) level 3 in DECT and SECT). Mixed DECT (linear blending of the images at two tube voltages) images were also reconstructed. The noise power spectrum and task transfer function of each scan protocol were quantified; the detectability index for each protocol was also determined using in‐house implementations of model observers (non‐prewhitening matched filters with internal noise, NPWI, and with an eye filter and internal noise, NPWEI) and human observers (in‐house four‐alternative forced choice, scoring with 95% confidence intervals).

**Results:**

Results show that the image noise is minimized at a VMI energy corresponding to the applied spectrum's mean energy in PCCT and with VMI settings of 70 and 80 keV for 70/150Sn and 80/150Sn tube potential pairs, respectively, in DECT. With respect to the human observer detectability index calculations, the normalized root‐mean‐square error for the NPWI and NPWEI model observers was 5% and 12%, respectively. PCCT VMI improves low‐contrast detectability. Additionally, detectability can be matched between PCCT protocols by increasing the QIR strength level when reducing the dose. Not only does PCCT VMI outperform DECT VMI, but also DECT VMI outperforms DECT mixed imaging in improving low‐contrast detectability.

**Conclusions:**

Low‐contrast detectability is optimized when the appropriate VMI energy level is selected in PCCT and DECT to minimize image noise. PCCT improves low‐contrast detectability and may allow for dose reduction in abdominal protocols compared to both DECT and SECT. The non‐prewhitening model observer with internal noise better quantified low‐contrast detectability without the inclusion of an eye filter.

## 1 INTRODUCTION

An accurate and clear representation of anatomy is essential for the assessment of pathology in clinical computed tomography (CT). At the same time, radiation dose should be minimized, considering that there is a trade‐off between patient dose and image quality. Balancing the aims of reducing image noise and improving image quality can be difficult, particularly in low‐contrast situations. In such situations, it may be difficult to distinguish a signal of interest from its background. Furthermore, the detectability of low‐contrast signals may vary due to differences in the imaging performance of different protocols and CT systems.

Until recently, clinical CT systems, including both dual‐energy CT (DECT) and single‐energy CT (SECT), have relied on energy‐integrating detectors, which allow for spatial discrimination of attenuation. However, photon‐counting CT (PCCT) systems rely on photon‐counting detectors, which allow for consideration of both the spatial and energy dependence of x‐ray attenuation in materials.^1^ Previous studies have shown that the spatial and energy discrimination that is available in PCCT may lead to improvements in the contrast‐to‐noise ratio (CNR),^2^ spatial resolution,^1^ dose efficiency^3^, ^4^ and image noise.^2^, ^3^ With the introduction of PCCT systems and more advanced iterative reconstruction (IR) algorithms in the clinic, there is potential to reduce examination doses without compromising image quality.

Typically, dose and image quality optimization are facilitated by physicists while radiologists perform visual assessments to evaluate the resulting image quality. However, this nonquantitative approach can be time‐consuming and can lead to inconsistencies in image quality across scanners and reconstruction algorithms. In order to improve the optimization of clinical CT protocols and to compare CT systems objectively, more quantitative methods for image quality evaluation can be implemented. Such methods include radiologists assessing image quality in receiver operating characteristic analysis, multiple alternative forced choice (M‐AFC) evaluations, and visual grading. However, these methods can be impractical to implement widely, as they can also be time‐consuming. Consequently, more practical approaches are also needed for the assessment of image quality.

Additionally, image quality evaluation of more complex imaging systems, like CT systems, poses difficulties due to the nonlinear nature of image processing techniques and IR algorithms. As a result, common methods to measure the imaging performance of radiological systems, such as the modulation transfer function (MTF),^5^ have limited utility in CT systems, which may have contrast‐dependent spatial resolution. For this reason, task‐based approaches, such as model observers (MO), together with the task transfer function (TTF)^6^ and noise power spectrum (NPS), can be used to estimate the performance efficiency of human observers for a specific diagnostic task in CT.^7^

Although MO can be used objectively to optimize CT protocols efficiently, it is necessary to begin by assessing their performance compared to that of human observers in real CT images. However, once they are validated for a set of protocols, MO have the potential to be generalized (i.e., not scanner or reconstruction algorithm specific) to optimize additional CT protocols. Due to the potential to improve visualization of signals in PCCT, the scope of this study was to assess low‐contrast detectability in PCCT, DECT, and SECT abdominal protocols using both M‐AFC and MO evaluations over various acquisitions settings, such as tube voltage and dose, and reconstruction settings, such as virtual monoenergetic imaging (VMI) energy (where applicable) and IR strength. Comparing various settings in clinical abdominal protocols, this work investigates avenues for optimizing low‐contrast detectability and also compares image quality between PCCT, DECT, and SECT.

## 2 METHODS

### 2.1 Phantom

**FIGURE 1:**
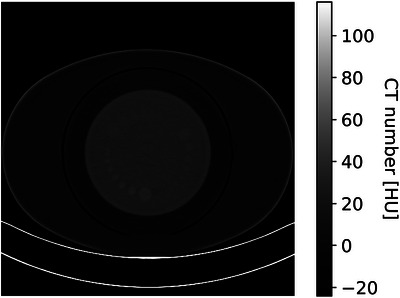
Ensemble average image of the CTP515 module of the Catphan 600 phantom nested with the CTP 579 oval annulus to simulate an abdomen with a water equivalent diameter of 298 mm.

A quality assurance phantom (Catphan 600, The Phantom Laboratory, Salem, New York, USA) was outfitted with an oval annulus (CTP 579, The Phantom Laboratory, Salem, New York) to mimic the attenuation of a patient's abdomen. The CTP486 image uniformity module was used for VMI selection experiments, and the CTP515 low‐contrast module with the 1% contrast supra‐slice targets (ϕ 4, 6, 9, and 15 mm) was used for all subsequent experiments (see Figure 1).

#### 2.1.1 Image acquisition and reconstruction

Imaging was performed on a first‐generation clinical dual‐source PCCT (NAEOTOM Alpha, VA50, Siemens Healthineers, Forchheim, Germany) in the single‐source, multi‐energy mode (Quantum and Quantum Plus) and a clinical dual‐source CT (SOMATOM Force, VB20, Siemens Healthineers, Forchheim, Germany) in both the dual‐source and single‐source mode for DECT and SECT. Spiral factory abdomen protocols were used without automatic tube voltage and current modulation to keep the CT dose index (${\mathrm{CTDI}}_{\mathrm{vol}}$) constant. On the PCCT system, images were acquired with all available tube voltages (70, 90, 120, and 140 kV) and seventeen VMI energies ranging between 40 and 100 keV. Certain VMI energies were chosen to include the mean photon energy of each applied spectrum. PCCT acquisitions were performed with the Quantum mode (“Quantum,” Siemens Healthineers) for 70 and 90 kV tube voltage settings, and the Quantum Plus mode (“Quantum plus,” Siemens Healthineers) for 120 and 140 kV tube voltage settings. On the DECT system, images were acquired with two tube voltage pairs (70/150Sn and 80/150Sn kV), which are used for abdominal protocols at our institution, and nine VMI energies ranging between 40 and 100 keV. Mixed DECT (linear blending of the images at two tube voltages) images were also generated. The SECT tube potentials were 100 and 120 kV. Hereafter, default settings for DECT and SECT will refer to a ${\mathrm{CTDI}}_{\text{vol}}$ of 10 mGy and ADMIRE level 3, which are typically used for abdominal protocols.

PCCT images were reconstructed with no quantum IR (QIR) (hereafter, referred to as level 0 or QIR 0) and all QIR strengths (hereafter, referred to as level 1–4 or QIR 1–4),^2^ where QIR 4 is the default and clinically used strength in abdominal protocols at our institution. Both DECT and SECT images were reconstructed with the default advanced modeled IR (ADMIRE) strength, where level 3 is the default. Hereafter, default settings for PCCT will refer to a ${\mathrm{CTDI}}_{\text{vol}}$ of 10 mGy and QIR level 4, which are typically used for abdominal protocols. All images were reconstructed with a Br40 kernel, a field‐of‐view of 350 mm, and 1.0 mm slice thickness; a slice increment of 0.7 and 1.0 mm were used for PCCT and DECT/SECT, respectively. Only one scan per protocol configuration was obtained for VMI optimization experiments, which only necessitated noise texture calculations. For experiments involving resolution and detectability calculations, every protocol configuration scan was repeated 66 times in PCCT, DECT, and SECT to achieve sufficient CNR for resolution calculations. The scan and reconstruction parameters are shown in Table 1.

**TABLE 1:** Summary of experimental image acquisition and reconstruction settings.

	Tube voltage (kV)	Current time product (mAs)	Rotation time (ms)	${\mathrm{CTDI}}_{\mathrm{vol}}$ (mGy)	Pitch	Acquisition (mm)	IR
PCCT	70	650	500	10	0.8	144 ×0.4	QIR 4
	90	281	500	10	0.8	144 ×0.4	QIR 4
	120	63	500	5	0.8	144 ×0.4	QIR 2, 4
	120	127	500	10	0.8	144 ×0.4	QIR 0‐4
	140	87	500	10	0.8	144 ×0.4	QIR 4
DECT	70/150Sn	494/124	500	10	0.6	128 ×0.6	ADMIRE 3
	80/150Sn	278/139	500	10	0.6	128 ×0.6	ADMIRE 3
SECT	100	249	500	10	0.6	192 ×0.6	ADMIRE 3
	120	150	500	10	0.6	192 ×0.6	ADMIRE 3

*Note*: PCCT acquisitions were performed with the Quantum mode (“Quantum,” Siemens Healthineers)for 70 and 90 kV tube voltage settings and the Quantum Plus mode (“Quantum plus,” Siemens Healthineers) for 120 and 140 kV tube voltage settings.

Abbreviations: DECT, dual‐energy computed tomography; PCCT, photon‐counting computed tomography; SECT, single‐energy computed tomography.

### 2.2 Image preprocessing for image quality analysis

The images from each scan were loaded and analyzed using Python 3 (Python Software Foundation) and the Pydicom library.^8^ A custom‐made script sorted all data and excluded the first and last five slices in each scan to ensure that no slices containing the module edge were included in subsequent resolution, noise texture, and detectability index analysis. This resulted in 40 and 36 usable slices per scan to calculate the image quality metrics in PCCT and DECT/SECT, respectively.

### 2.3 Image quality analysis: NPS

The NPS was assessed to quantify image noise texture and magnitude. Two regions‐of‐interest (ROI) of size 24 × 24 pixels or about 16 × 16 mm were placed along the uniform part of the phantom, and the NPS was calculated. Small ROIs were used for NPS calculation to obtain the spatial correlations with minimal loss of information from averaging processes and to more closely approximate the local wide‐sense stationary noise, considering the small size of the simulated clinical tasks and actual phantom target sizes of interest.^9^ NPS calculations were made with a larger ROI of size 30 × 30 pixels, yielding an NPS in close agreement with that of the smaller ROI. This comparison was made to verify that the results were not sensitive to the ROI size. The 2D‐NPS was calculated as follows:

(1) $$\text{NPS}\left(u,v\right)=\frac{\Delta x\Delta y}{n_{x}n_{y}}\left\langle \right|{\text{FFT}}_{2D}\left[I\left(x,y\right)-\bar{I}\left(x,y\right)\right]{{|}^{2}\rangle }_{n},$$

where Δx and Δy are the pixel sizes (in mm) in both image directions, $n_{x}$ and $n_{y}$ are the ROI sizes (the number of pixels in the image), ${\text{FFT}}_{2D}$ refers to the 2D fast Fourier transform, I(x,y) is the ROI pixel value, $\bar{I}\left(x,y\right)$ is the background pixel‐value, and n is the number of ROIs.

Noise magnitude or pixel variance was measured by integrating the 2D‐NPS and averaging over usable slices in all scans. For each protocol configuration, the 2D‐NPS was averaged over useable slices in all scans, radially binned, and averaged to a 1D‐NPS curve, using the NPS scripts from the ImQuest open‐source repository.^7^ ImQuest's NPS‐scripts were modified to accommodate our dataset. In VMI optimization experiments, the total number of NPS ROIs was 200 (1 scan× 40 slices× 5 ROIs per slice) in PCCT and 180 (1 scan×36 slices×5 ROIs per slice) in DECT/SECT. In experiments involving resolution and detectability calculations, the total number of NPS ROIs was 5280 (66 scans× 40 slices×2 ROIs per slice) in PCCT and 4752 (66 scans×36 slices×2 ROIs per slice) in DECT/SECT. The peak spatial frequency $f_{\text{peak}}$ was determined at the maximum of the NPS.

### 2.4 Image quality analysis: TTF

The TTF was assessed to quantify contrast‐dependent spatial resolution of the systems. The TTF is assumed to be contrast dependent and size independent,^6^ and as such, the 15 mm (i.e., largest) contrast target in the phantom was used for TTF calculations to ensure sufficient sampling. The TTF was calculated for every protocol and system, using the circular‐rod method^6^ implemented in the TTF‐scripts from the ImQuest open‐source repository.^7^ ImQuest's TTF‐scripts were modified to accommodate our dataset.

Split data acquisitions (i.e., repetitions were acquired in multiple sittings) were necessary due to incoming emergency patients. The repetitions in a single reposition event were averaged in a slice‐by‐slice fashion to create an ensemble average image. The TTF was calculated for each slice in this ensemble average image, and the resulting TTFs for each slice were averaged to create the ensemble average TTF. This was done for each reposition event, and the final TTF was calculated by averaging the ensemble average TTF of all reposition events. This approach was pursued to optimize the use of the available slices, to avoid issues with repositioning, and to avoid resolution degradation/noise in the TTF calculation if inserts were not exactly parallel to the *z*‐axis of the CT systems. The number of scan repeats and usable slices (66 scans× 40 slices in PCCT and 66 scans × 36 slices in DECT/SECT) were estimated to yield a combined CNR≥11 per protocol configuration, where a CNR of 15 has been estimated to result in a 10% error.^10^ The frequency in which the TTF dropped to 50%, $f_{50}$, was extracted from TTF calculations to quantify resolution.

### 2.5 Image and statistical analysis: M‐AFC human observations

**FIGURE 2:**
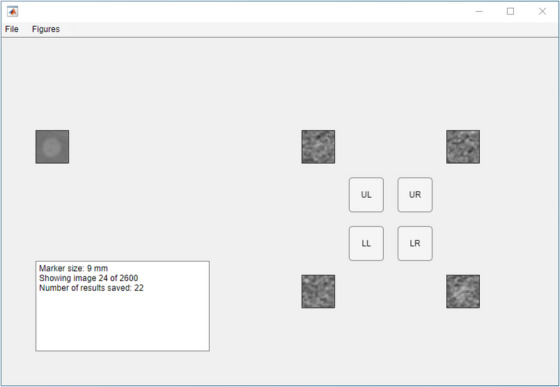
In‐house M‐AFC user interface. M‐AFC, multiple alternative forced choice.

To measure observer‐based imaging performance in PCCT protocols, M‐AFC tests were performed on a radiological monitor with phantom images that were acquired with a subset of the PCCT protocols listed in Table 1. A graphical user interface was constructed using MATLAB (R2022a, The MathWorks Inc., Massachusetts, United States) to display ROIs from the phantom images and collect observer responses. An example of the user interface is shown in Figure 2. Observers included four physicists and two radiologists. They were required to identify which of M=4 ROIs contained the circular signal, where M−1 images contained no signal or the background, and one image contained the signal. The ROIs with the signal contained one of the supra‐slice circular targets of interest (ϕ 4, 6, and 9 mm) in the phantom. Background ROIs consisted of a background near the target of interest in the phantom.

The radiological monitor was 432 mm × 325 mm in size and had a resolution of 2048 × 1536 pixels, resulting in an approximate pixel size of 0.21 mm. Each image pixel was shown on 2 × 2 monitor pixels, and the distance between the observer's eyes and monitor was estimated to be 50 cm. For three target sizes (ϕ 4, 6, and 9 mm), ROIs were cropped from axial slices of a 3D reconstruction with the signal at the center of the ROI. Noise ROIs were cropped to the same size and taken from the same slice surrounding the signal of interest. Target size‐dependent ROI sizes are listed in Table 2.

**TABLE 2:** M‐AFC signal visualization settings.

Target size (mm)	Image ROI size (pixels)	Monitor ROI size (pixels)	Monitor ROI size (mm)
4	14 ×14	28 ×28	5.9 ×5.9
6	20 ×20	40 ×40	8.4 ×8.4
9	24 ×24	48 ×48	10.1 ×10.1

Abbreviations: M‐AFC, multiple alternative forced choice; ROI, regions of interest.

Additionally, different display window settings were necessary to visualize the target signals because various PCCT protocols with differing tube potentials were used in M‐AFC tests. For all tube voltages, a window width of 140 HU was used. The following window centers were used: for 70 kV, 21 HU; for 90 kV, 46 HU; for 120 kV, 54 HU; and for 140 kV, 57 HU. Window centers were chosen as the average of the CT numbers of the feature and background within the displayed subimages.

A figure of merit for task performance is given by the fraction of correct responses $P_{\text{corr}}$ and can be related to the detectability index $d_{\text{AFC}}^{\prime }$, using tabulated values.^11^ Assuming that an observer randomly chose the signal present image in all M‐AFC tasks with M=4, a value of $P_{\text{corr}}=25\%$ was expected and as such, was used as the limit of allowable $P_{\text{corr}}$ in the M‐AFC tests. Data from one observer was excluded from the analysis due to large deviations of $P_{\text{corr}}$ for all protocols compared to results from other observers and because in most protocols $P_{\text{corr}}$ was below 25%.

The standard error of $d_{\text{AFC}}^{\prime }$ was calculated as the sample standard deviation of $d_{\text{AFC}}^{\prime }$ over all included observers for a given imaging protocol divided by the square root of the number of observers. The standard error of $d_{\text{AFC}}^{\prime }$ was then used to determine 95% confidence intervals of $d_{\text{AFC}}^{\prime }$.

### 2.6 Image quality analysis: MO and the detectability index

Overall image quality for the low‐contrast targets was assessed with MO using the detectability index $d^{\prime }$, which represents how well a signal can be detected from information about both noise texture and spatial resolution of the system. Various forms of a non‐prewhitening matched filter (NPW) model observer were investigated to characterize the detectability index. In‐house MATLAB scripts were used to perform all model observer calculations.

Many studies^12^, ^13^ have shown that the NPW observer correlates well with the detection accuracy of human observers in CT, which is a modality for which humans have demonstrated little evidence of being able to pre‐whiten noise. The basic NPW model observer has the following form:^14^

(2) $$\begin{array}{ccc} & & {\left(d_{\text{NPW}}^{\prime }\right)}^{2}\\ & & \;=\frac{\left[\int \int \right|W_{\text{task}}{{|}^{2}\cdot {\text{TTF}}^{2}\left(u,v\right)\;dudv]}^{2}}{\int \int |W_{\text{task}}{\left(u,v\right)|}^{2}\cdot {\text{TTF}}^{2}\left(u,v\right)\cdot \text{NPS}\left(u,v\right)\;dudv}, \end{array}$$

where $d_{\text{NPW}}^{\prime }$ is the detectability index for the NPW model observer, $W_{\text{task}}\left(u,v\right)$ is the task function or the Fourier transform of the signal of interest, and uandv are spatial frequencies in ${\mathrm{mm}}^{-1}$. In this work, circular and uniform objects with rectangular contrast profiles were assumed as the signal of interest. Signals with a contrast of 10 HU and diameters between 3 and 10 mm were calculated.

The NPW observer can be extended to include an eye filter (NPWE)^15^ as follows:

(3) $$\begin{array}{ccc} & & d_{\text{NPWE}}^{\prime 2}\\ & & \;=\frac{\left[\int \int \right|W_{\text{task}}{{|}^{2}\cdot {\text{TTF}}^{2}\left(u,v\right)\cdot E^{2}\left(u,v\right)\;dudv]}^{2}}{\int \int |W_{\text{task}}{\left(u,v\right)|}^{2}\cdot {\text{TTF}}^{2}\left(u,v\right)\cdot \text{NPS}\left(u,v\right)\cdot E^{4}\left(u,v\right)\;dudv}, \end{array}$$

where $d_{\text{NPWE}}^{\prime }$ is the detectability index for the NPWE model observer, and E(u,v) is the eye filter. The eye filter is often introduced to model the effect of the contrast sensitivity function of the human visual system, which suppresses low spatial frequencies as a result of retinal processing. Various forms of E(u,v) exist in the literature.^15^, ^16^, ^17^ In this paper, a commonly used eye filter is employed,^a^ based on one proposed by Eckstein et al.,^16^

(4) $$E\left(\rho \right)={\left(\rho^{a}\exp\left(-b\;\rho^{c}\right)\right)}^{2},$$

where a=1.5, b=0.98, and c=0.68 and the peak frequency response is 3.3 cycles/degree. The radial spatial frequency in cycles per degree, ρ, is calculated as follows:^16^

(5) $$\rho =\frac{\text{FOV}\cdot R\cdot \pi }{180\cdot D}\omega ,$$

where $\omega^{2}=u^{2}+v^{2}$, FOV=350 mm is the reconstruction FOV, D=215 mm is the display size, and R=500 mm is the viewing distance.

The NPW and NPWE observer can be further degraded with internal noise (NPWI and NPWEI, respectively) as follows:

(6) $${\left(d_{\text{NPWI}/\text{NPWEI}}^{\prime }\right)}^{2}=\frac{1}{\left(1+\beta \right)}{\left(d_{\text{NPW}/\text{NPWE}}^{\prime }\right)}^{2},$$

where β is the dimensionless coefficient of induced image noise. The subscripts in Equation (6) refer to the scaling of $d_{\text{NPW}}^{\prime }$ and $d_{\text{NPWE}}^{\prime }$ to yield $d_{\text{NPWI}}^{\prime }$ and $d_{\text{NPWEI}}^{\prime }$, respectively. The detectability indices $d_{\text{NPWI}}^{\prime }$ and $d_{\text{NPWEI}}^{\prime }$ are for the NPWI and NPWEI MO, respectively. Internal noise is often introduced to model the efficiency of neural mechanisms that handle signal discrimination in the human visual system.^12^, ^18^ Previous studies have shown that fixed internal noise only dominates when the image noise is difficult to perceive^19^ and that in most cases, “induced noise” dominates, with β treated as a constant.^16^ In this paper, we only consider induced internal noise as in the work of Ishida et al.^18^ and treat β as a free parameter in fitting NPW‐based MOs to M‐AFC data. Our work investigates the performance of both the NPWI and NPWEI MOs in Equation (6) with the commonly used version of the Eckstein filter in Equation (4).

### 2.7 VMI selection in PCCT and DECT

Low‐contrast rods and the background material in the phantom have closely equivalent effective atomic numbers, and only the mass density is varied to produce changes in the effective attenuation coefficients. As a result, noise is the driving factor for the detectability of the low‐contrast targets in the phantom. A VMI energy, corresponding to the applied spectrum's mean energy, is used by default in factory PCCT protocols. Mixed images are used by default in factory DECT protocols. Therefore, VMI energy selection was investigated to minimize image noise in both PCCT and DECT.

The NPS and associated pixel variances were calculated over a range of VMI energy and tube voltage settings as described in Section 2.1.1. Default ${\mathrm{CTDI}}_{\mathrm{vol}}$ and IR settings were used. This selection was performed for various tube voltage settings in PCCT and DECT, which are listed in Table 1. VMI settings that minimized image noise (i.e., optimal VMI settings) for each tube potential and tube potential pair were used in subsequent low‐contrast detectability investigations in PCCT and DECT, respectively.

### 2.8 Noise texture and resolution in abdominal PCCT protocols

Optimal VMI settings were used to investigate noise texture and resolution in PCCT. Investigations were performed on the PCCT tube voltages with default ${\mathrm{CTDI}}_{\mathrm{vol}}$ and QIR settings. Additional investigations for a tube potential of 120 kV were performed with settings of 5 mGy with QIR levels 2 and 4; and 10 mGy with QIR level 2. The NPS, pixel variance, TTF, and $f_{50}$ were calculated to determine noise and resolution differences between protocols.

### 2.9 Validation and optimization of the NPWEI MO in abdominal PCCT protocols

For the same PCCT protocols in Section 2.8, M‐AFC observations were performed the corresponding detectability indices $d_{\text{AFC}}^{\prime }$ (averaged over five observers) were calculated and used to fit both the NPWI and NPWEI MOs. The $d^{\prime }$ of the MOs were calculated on the same phantom images as used in the M‐AFC observations. In‐house MATLAB scripts were used for fitting and a nonlinear least squares solution was pursued to minimize the sum of the squares of the differences between MO and M‐AFC detectability index values with respect to β in Equation (6). The *fmincon* MATLAB function was used for the optimization and to constrain the fit with β>0. To quantify the performance of the MOs, the normalized root mean square error (nRMSE) between the $d^{\prime }$ of the MO and $d_{\text{AFC}}^{\prime }$ over all protocols in Section 2.8 was calculated (i.e., RMSE divided by the range of $d_{\text{AFC}}^{\prime }$ values). The fitted parameter was used to determine efficiency of an average observer, which could be applied to additional PCCT, DECT, and SECT protocols. Furthermore, the fit was repeated on an individual basis to determine the efficiency of individual observers.

### 2.10 Image quality in abdominal PCCT, DECT, and SECT protocols

Noise texture and resolution metrics were calculated for PCCT, DECT, and SECT abdominal protocols with various tube voltage settings and optimal VMI settings in PCCT and DECT. Investigations were performed on all protocols with default ${\mathrm{CTDI}}_{\mathrm{vol}}$ and IR settings. The detectability index was calculated using the fitted β parameter of an average observer.

## 3 RESULT

### 3.1 Optimization of VMI in PCCT and DECT

**FIGURE 3:**
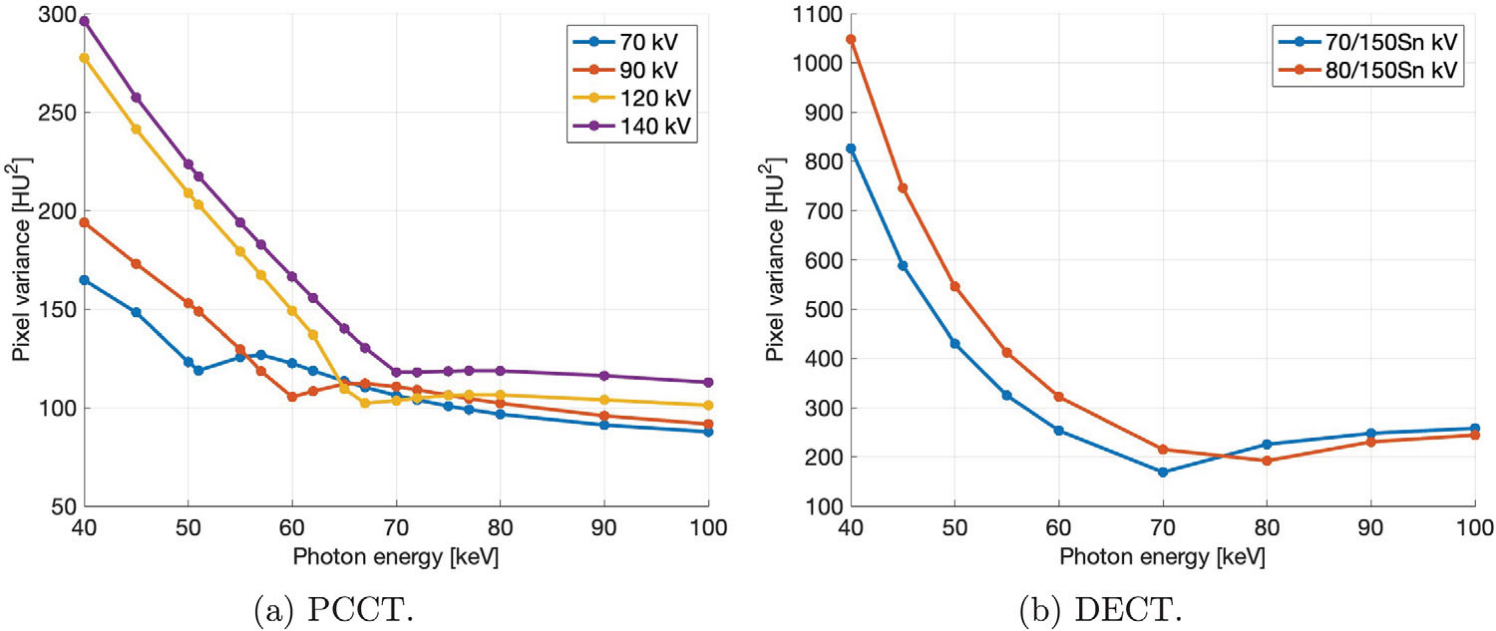
Pixel variance as a function of virtual monoenergetic photon energy in (a) PCCT and (b) DECT. The scale on the vertical axes in (a) and (b) differ. DECT. dual‐energy computed tomography; PCCT, photon‐counting computed tomography.

The NPS for PCCT and DECT protocols over various VMI energies are shown in the supplementary material section (see Figure S1). In PCCT, image noise (i.e., pixel variance) was minimized at the first local minimum in Figure 3a, which corresponded to images that were reconstructed with a VMI energy corresponding to the applied spectrum's mean energy (i.e., 51 keV for 70 kV, 60 keV for 90 kV, 67 keV for 120 kV, and 70 keV for 140 kV). In DECT, image noise was minimized with VMI settings of 70 keV and 80 keV for 70/150Sn kV and 80/150Sn kV tube potential pairs, respectively (see Figure 3b).

### 3.2 Noise texture and resolution in abdominal PCCT protocols

**FIGURE 4:**
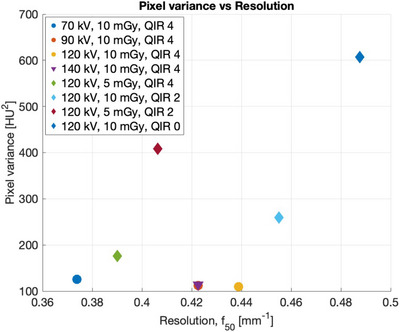
For various tube voltages in PCCT, pixel variance versus $f_{50}$ is shown. Note that the points for the 90 and 140 kV protocols overlap. QIR 0 results are shown for reference but were not used for the fitting of the NPWI MO. MO, model observer; NPWI, non‐prewhiteing matched filters with internal noise; PCCT, photon‐counting computed tomography; QIR, quantum iterative reconstruction.

The NPS, ESF, and TTF for PCCT protocols are shown in the supplementary material section (see Figure S2). The NPS peaked between 0.11 and 0.16 ${\mathrm{mm}}^{-1}$, where the highest peak frequency values corresponded to protocols using the lower QIR strength. For the standard protocol (i.e., 120 kV, 10 mGy, QIR 4), the peak frequency was 0.14 ${\mathrm{mm}}^{-1}$. Each tube voltage in PCCT was associated with different pixel variances and $f_{50}$ values (see Figure 4). The TTF had $f_{50}$ values between 0.37 and 0.46 ${\mathrm{mm}}^{-1}$. Based on the standard protocol, the TTF had an $f_{50}$ value of 0.44 ${\mathrm{mm}}^{-1}$. Protocols using a lower QIR level of 2 and a typical tube voltage setting of 120 kV were associated with larger pixel variances and $f_{50}$ values. Generally, protocols using a lower CTDIvol setting of 5 mGy had larger pixel variances and lower $f_{50}$ values. Protocols using tube voltage settings between 90 to 140 kV with a default CTDIvol of 10 mGy and QIR level 4, had the lowest pixel variances.

### 3.3 Fitting of the NPWI MO in abdominal PCCT protocols

**FIGURE 5:**
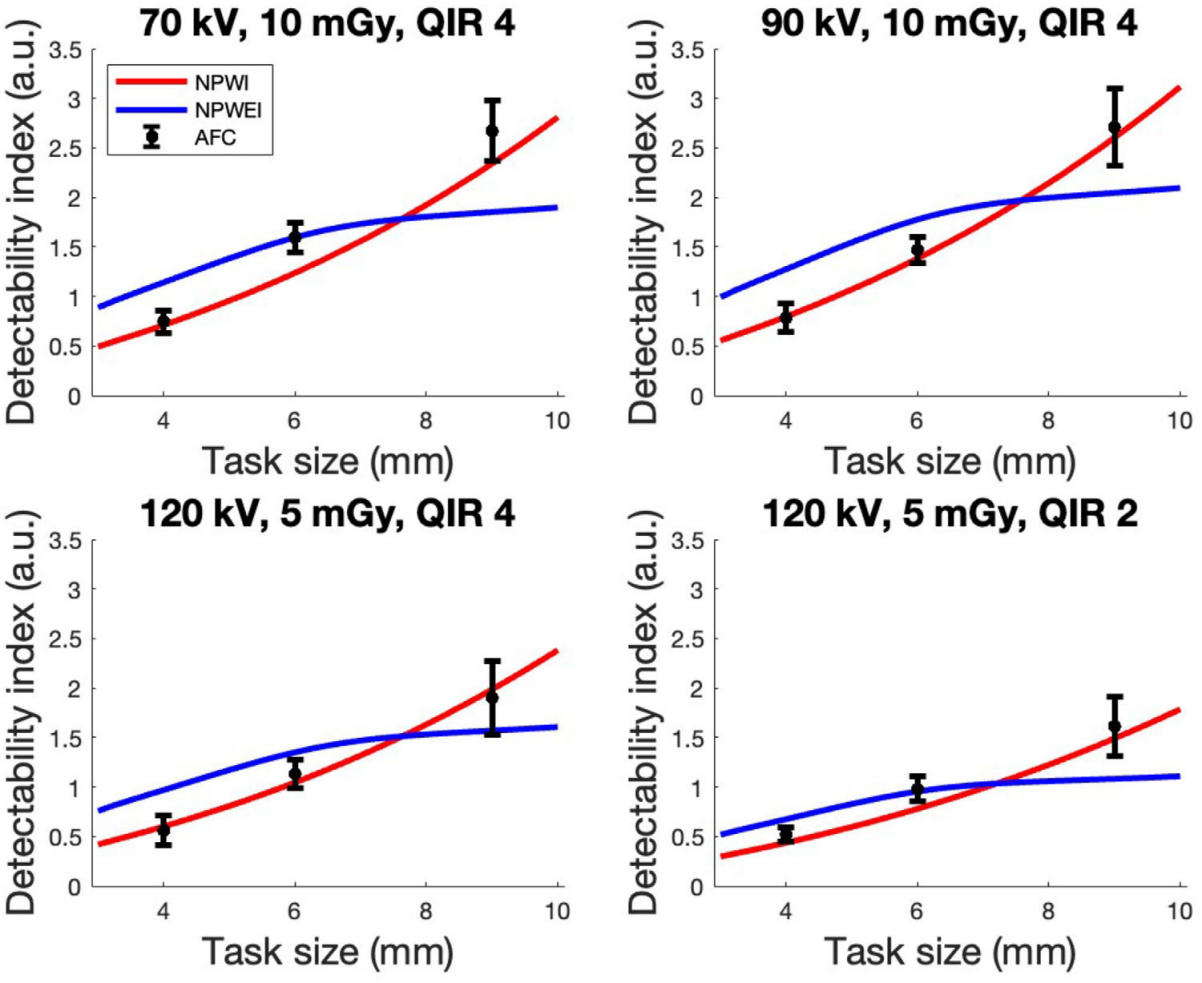
NPWI, NPWEI, and M‐AFC detectability index of various PCCT protocols as a function of task size for fitting. The NPWI and NPWEI MO were fitted with respect to M‐AFC evaluations. The error bars on M‐AFC points correspond to 95% confidence intervals. M‐AFC, multiple alternative forced choice; MO, model observer; NPWI, non‐prewhiteing matched filters with internal noise; NPWEI, non‐prewhiteing matched filters with an eye filter and internal noise; PCCT, photon‐counting computed tomography; QIR, quantum iterative reconstruction.

For a selection of protocols used in the fitting, detectability index calculations for the fitted NPWI MO, fitted NPWEI MO, and M‐AFC observations are shown in Figure 5. Results show that the optimization yields an overall nRMSE of 5% and 12% with the NPWI MO and NPWEI MO, respectively. Optimization of the NPWI MO yielded an optimized internal noise parameter for an average observer of β=7.1. The coefficients of induced image noise of individual observers are shown in Table 3. Generally, β decreased with the level of observer experience and was the lowest for a radiologist.

**TABLE 3:** Coefficients of induced image noise of individual observers derived from optimization of the β parameter in the internal noise of the NPWI MO in Equation (6).

Observer	β
Average observer	7.1
Radiologist	4.7
Physicist 1	8.9
Physicist 2	7.1
Physicist 3	11.3
Physicist 4	5.8

Abbreviations: MO, model observer; NPWI, non‐prewhiteing matched filters with internal noise.

### 3.4 Image quality in abdominal PCCT, DECT, and SECT protocols

**FIGURE 6:**
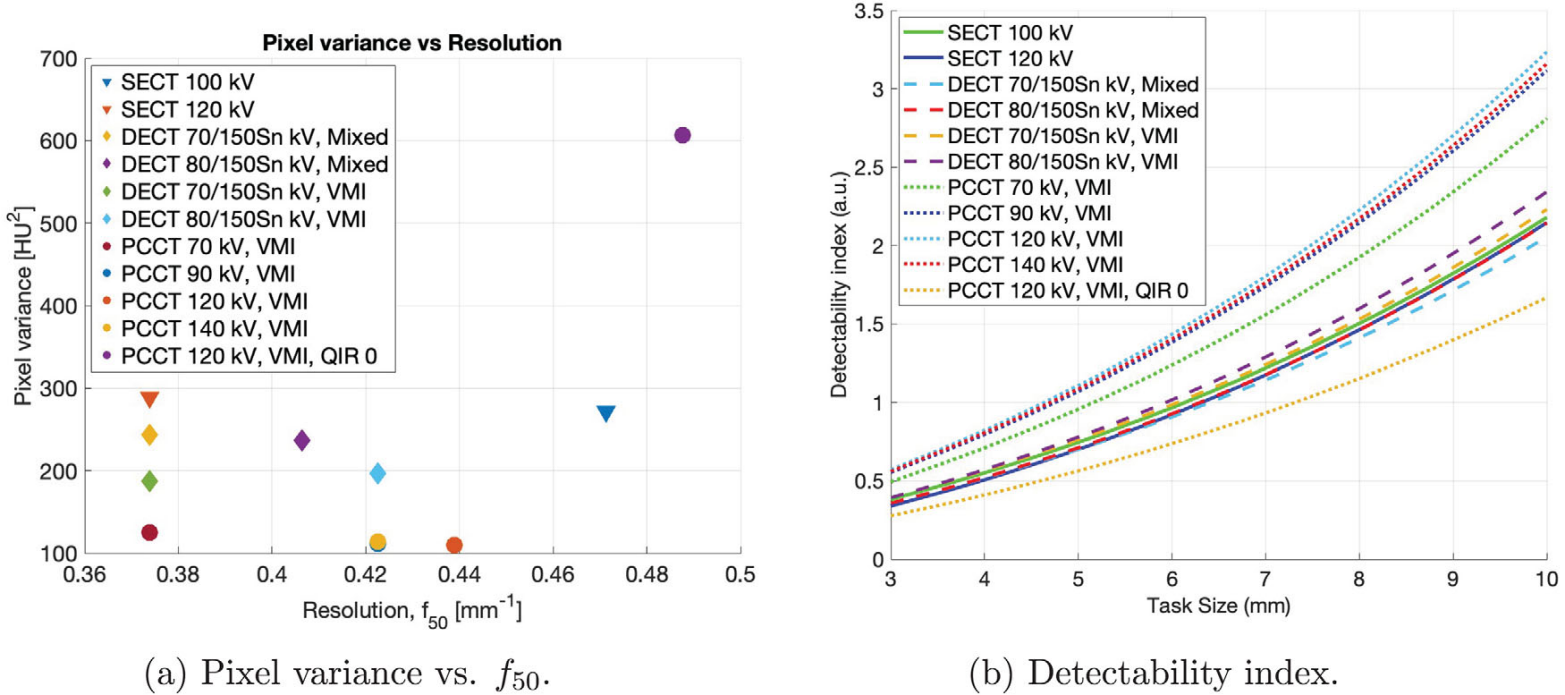
For various abdominal protocols in PCCT, DECT, and SECT, (a) pixel variance versus $f_{50}$ and (b) NPWI MO detectability index of abdominal PCCT, DECT, and SECT protocols are shown. Note that the points for the 90 and 140 kV PCCT protocols overlap in (a) and the lines for the SECT 120 kV and DECT 80/150Sn kV, Mixed protocols overlap for larger task sizes in (b).QIR 0 results are shown for comparison. DECT, dual‐energy computed tomography; MO, model observer; NPWI, non‐prewhiteing matched filter with internal noise; PCCT, photon‐counting computed tomography; QIR, quantum iterative reconstruction; SECT, single‐energy computed tomography.

The NPS and TTF for PCCT, DECT, and SECT protocols were calculated and are shown in the supplementary material section (see Figure S3). The NPS for abdominal PCCT, DECT, and SECT protocols peaked between 0.11 mm and 0.17 ${\mathrm{mm}}^{-1}$. Each protocol was associated with different pixel variances and $f_{50}$ values (see Figure 6a). The TTF for DECT protocols had $f_{50}$ values between 0.37 and 0.43 ${\mathrm{mm}}^{-1}$, where the lowest resolution was associated with protocols using a tube voltage setting of 70/150Sn kV. The TTF for SECT protocols had $f_{50}$ values between 0.37 and 0.47 ${\mathrm{mm}}^{-1}$. The largest pixel variances were associated with SECT protocols. For some protocols, $f_{50}$ values were comparable between PCCT, DECT, and SECT. For both VMI and mixed images in DECT, higher tube voltage in one of the sources yielded higher $f_{50}$ values. Generally, PCCT protocols were associated with the lowest pixel variances, and the QIR 0 setting was associated with the highest resolution.

The optimized NPWI MO for an average observer was used to quantify the detectability of the PCCT, DECT, and SECT protocols. Results are shown in Figure 6b. The lowest detectability was associated with SECT protocols and DECT protocols that used mixed images. Compared to SECT, slight improvements in detectability were seen with DECT protocols that used optimized VMI. However, the highest detectability of the low‐contrast signals can be achieved with protocols using tube voltages greater than 70 kV and optimized VMI in PCCT. Average observer NPWI detectability index values were calculated for additional PCCT protocols over all QIR strength levels and are shown in the supplementary material section (see Figure S4).

## 4 DISCUSSION

In conjunction with the implementation of clinical PCCT, an QIR was also introduced in 2021. Although previous studies have shown that PCCT improves CNR,^2^ spatial resolution,^1^ and image noise,^2^, ^3^ modern reconstruction algorithms, such as QIR, can often complicate the assessment of image quality. Therefore, it is necessary to use task‐based metrics in the assessment of image quality, particularly when noise texture, noise magnitude, and resolution may vary between imaging conditions.^7^ In this study, we evaluate low‐contrast detectability of abdominal protocols in PCCT, DECT, and SECT for a range of tube potentials, doses, IR strength levels, VMI energies, and target object size, in addition to providing a direct comparison of low‐contrast detectability of VMI in clinical PCCT and DECT. Our findings show four major results: (1) PCCT VMI can improve the detectability of low‐contrast features, (2) detectability can be matched between PCCT protocols by increasing the QIR strength level when reducing the dose, (3) not only does PCCT VMI outperform DECT VMI but also DECT VMI outperforms DECT mixed imaging in improving low‐contrast detectability, and (4) the NPWI MO with induced internal noise can predict human performance on the detectability of low‐contrast signals over a wide range of protocols.

Various groups have investigated noise texture^2^, ^20^ and iodine detectability^21^, ^22^ in the same clinical PCCT system with QIR and detectability in the corresponding prototype PCCT system with FBP‐like reconstruction.^4^, ^23^, ^24^ Generally, most studies on both clinical and prototype systems investigated only PCCT and SECT, except for one prototype PCCT system study,^24^ which also investigated DECT. Sartoretti et al.^2^ calculated NPS for PCCT images reconstructed with QIR and also found that there is a decrease in image noise with increasing QIR strength and that the peak NPS frequency for a standard protocol (i.e., 120 kV) using the highest QIR strength has a peak frequency of 0.14 mm 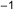. One study has found that for mixed images in PCCT, the peak frequency of the NPS is constant for all QIR strengths.^20^ However, our study shows a slight shift toward lower peak frequencies with increasing QIR strength with VMI images. Nonetheless, our investigations show that the peak frequency of the NPS remains relatively constant over tube voltage, dose, and VMI setting in PCCT and even DECT/SECT in acquisitions using the same reconstruction kernel and dose. Our study also confirms that the $f_{50}$ for the TTF of FBP‐like reconstruction (i.e., QIR 0) is higher than that of IR (i.e., higher levels of IR) as shown in the work of Richard et al.^6^ Differences in TTF measurements may be seen when different reconstruction methods (IR algorithms and/or kernels) and phantoms (insert composition and contrast) are used.^6^, ^10^ For a reconstruction kernel similar to the Br40 kernel used in this study (i.e., Siemens body kernel), the TTF may show overshooting in the low‐frequency range depending on the contrast of the task of interest and the dose level,^4^ especially at low doses. Our study focused on very low‐contrast tasks with a nominal contrast of 1% or about 10 HU and used a nonedge‐enhancing reconstruction kernel with a moderate dose level. Our TTF measurements did not show this overshooting effect. When using the standard resolution scan mode and the Br40 reconstruction kernel, our work also confirms that image noise decreases with increasing QIR strength and further extends this observation to show that detectability increases.

Of the studies on similar clinical PCCT systems, one study assessed the image quality of high‐contrast objects as a function of the reconstruction kernel and phantom size in both PCCT and SECT.^21^ This group used the NPW observer in the ImQuest software^7^ to quantify detectability and reported that PCCT showed better noise and contrast than SECT for the same reconstruction kernels at various doses and patient sizes. In low‐dose acquisitions, this group also reports that PCCT outperformed SECT in terms of noise and CNR. Our investigations also show that in low‐contrast objects, PCCT shows lower image noise and improved detectability than SECT for the same reconstruction kernels and various tube voltage settings at a standard abdominal CT dose. We also extend these results to show the same trend between PCCT and DECT. Another study used a channelized Hotelling observer to evaluate low‐contrast detectability in both PCCT and SECT.^22^ This group reported that PCCT showed similar and higher detectability than SECT when no IR and IR, respectively, were used to reconstruct low‐energy threshold images. Our investigations also demonstrate that PCCT shows higher detectability than SECT when IR is used. We also extend these results to show that PCCT shows higher detectability than DECT for various tube voltages and when IR and VMI are used to reconstruct.

Of the studies that have also quantified detectability with NPW‐based MO in a prototype PCCT^4^ and a clinical PCCT system,^21^ implementation of the MO has differed. Bhattarai et al.^21^ only applied an NPW MO and human observer assessments of image quality were not made. Rajagopal et al.^4^ employed the free parameters values for internal noise and the eye filter from previous studies without validating this NPWEI MO to the human observer. Although this kind of implementation of the NPWEI MO is not limited to PCCT studies,^25^, ^26^, ^27^, ^28^, ^29^ we note that it is beneficial to perform human observer assessments of image quality and to determine the internal noise and even the eye filter, if it is to be used, based on these observations. This is particularly important in new evaluations of the detectability of targets with different contrast and size than previous evaluations. Previously used internal noise and eye filter parameters may not reliably predict human observer performance if the imaging conditions relating to target contrast and size are not carefully considered.

Our investigations showed that the NPWEI MO used in studies of detectability in CT,^25^, ^26^, ^27^, ^28^, ^29^ which are based on parameters derived from older literature,^15^, ^16^, ^17^ did not, in this case, reliably predict observer performance from M‐AFC tests. Both an NPWI MO and NPWEI MO were used to investigate detectability; however, the utility of the eye filter is not entirely clear. CT noise is not low‐pass.^30^ Nonetheless, some studies have suggested that the usefulness of the NPWEI MO is related to the eye filter's ability to suppress low spatial frequency noise.^31^, ^32^, ^33^ Similarly, Richard et al.^26^, ^34^ suggested that anatomical background noise can further degrade the NPWEI MO at low spatial frequencies in a similar manner to the ramp‐like eye filter. Although eye filters and anatomical noise yield a similar effect of diminishing low spatial frequency performance, it is unclear how effects that originate from limitations in the human visual system or that depend on perception of image noise should be implemented in a MO.

Although various forms of the NPWEI MO have been implemented with some success, both the NPWI MO and the NPWEI MO can perform similarly with respect to M‐AFC observations,^25^ suggesting that the eye filter can possibly be neglected. In our investigations, we implemented a variety of eye filters^15^, ^16^, ^17^ without success. As a result, we neglected the eye filter, using an NPWI MO with induced internal noise treated as a free parameter to best fit our M‐AFC data.

This study has strengths and limitations. Strengths include the direct comparison of PCCT, DECT, and SECT in the same experiments, the use of several different acquisition (tube voltage, dose) and reconstruction parameters (IR strength and VMI levels), the use of multiple target sizes, the inclusion of M‐AFC observations, and the incorporation of an NPWI MO to best fit M‐AFC data. Limitations include that uniform backgrounds, uniform targets of a single contrast, and relatively large targets were evaluated, which may not reflect many realistic clinical tasks. Although only one phantom size was investigated in this work, the phantom used corresponded closely to water equivalent diameters used in quality control tests performed by medical physicists. As a further limitation, we assumed that induced internal noise dominates over fixed internal noise,^16^ and as such, the form of the NPWI and NPWEI MOs used in Equation (6) only included the former. Nonetheless, this assumption resulted in a model that fit M‐AFC observation data well.

In our assessment of image quality in PCCT, low‐contrast detectability was improved with higher tube voltages and showed that the detectability can be matched between protocols with different doses and IR strengths. That is, dose reduction in abdominal protocols may be possible with PCCT compared to both DECT and SECT. Furthermore, we have shown that not only does PCCT VMI outperform DECT VMI and SECT but also DECT VMI outperforms DECT mixed imaging and SECT in improving detectability of low‐contrast signals. These findings may assist in the optimization of PCCT imaging protocols to improve low‐contrast detectability of the abdomen and to encourage the use of VMI where it is not yet used routinely (e.g., DECT). Additionally, improvements in low‐contrast detectability could facilitate in earlier and more accurate diagnosis of abdominal pathologies, such as cysts, which may present with subtle contrast.^35^ Furthermore, improved detectability allows for the reduction of radiation doses without compromising image quality, which may be beneficial in pediatric and follow‐up imaging.

## 5 CONCLUSIONS

This study reports on phantom evaluations of a clinical first‐generation PCCT scanner with a focus on the detectability of low‐contrast targets of various sizes in abdominal protocols with a variety of tube voltages, doses, VMI energy levels, and IR strength settings. The non‐prewhitening model observer with internal noise better quantified low‐contrast detectability without the inclusion of an eye filter. Clinical PCCT produces images with lower noise and higher detectability than both DECT and SECT. PCCT may allow for dose reduction in abdominal protocols compared to both DECT and SECT. Improvements in low‐contrast detectability in the abdomen may aid in the assessment of pathologies in CT.

## CONFLICT OF INTEREST STATEMENT

One author is an employee of Siemens Healthineers Sweden; this author did not have control over the data at any point during the study.

## Supporting information

Supporting Information

Supporting Information

Supporting Information

Supporting Information

## Data Availability

The data used in this study is available upon request.

## FOOTNOTES

a It is common for a version of the Eckstein eye filter ^16^ to be employed in detectability studies in CT. This version includes the power of 2 shown in Equation (4). Although this version of the Eckstein eye filter has been employed with some success, the original Eckstein eye filter was implemented with a power of 1.

## References

[1] Taguchi K , Iwanczyk J . Vision 20/20: single photon counting x‐ray detectors in medical imaging. Med Phys. 2013;40(10):100901. 24089889 10.1118/1.4820371 PMC3786515

[2] Sartoretti T , Landsmann A , Nakhostin D , et al. Quantum iterative reconstruction for abdominal photon‐counting detector CT improves image quality. Radiol. 2022;303(2):339‐348. 10.1148/radiol.211931 35103540

[3] Liu LP , Shapira N , Chen AA , et al. First‐generation clinical dual‐source photon‐counting CT: ultra‐low‐dose quantitative spectral imaging. Eur Radiol. 2022;32(12):8579‐8587. 35708838 10.1007/s00330-022-08933-x PMC10071880

[4] Rajagopal JR , Farhadi F , Solomon J , et al. Comparison of low dose performance of photon‐counting and energy integrating CT. Acad Radiol. 2021;28(12):1754‐1760. 32855051 10.1016/j.acra.2020.07.033 PMC7902731

[5] Metz C , Doi K . Transfer function analysis of radiographic imaging systems. Phys Med Biol. 1979;24(6):1079‐1106. 394162 10.1088/0031-9155/24/6/001

[6] Richard S , Husarik D , Yadava G , Murphy S , Samei E . Towards task‐based assessment of CT performance: system and object MTF across different reconstruction algorithms. Med Phys. 2012;39(7):4115‐4122. 22830744 10.1118/1.4725171

[7] Samei E , Bakalyar D , Boedeker KL , et al. Performance evaluation of computed tomography systems: summary of AAPM task group 233. Med Phys. 2019;46(11):e735‐e756. 31408540 10.1002/mp.13763

[8] Pydicom : pydicom: An open source DICOM library . Accessed May 20, 2022. www.pydicom.github.io/pydicom/dev

[9] Dolly S , Chen H , Anastasio M , Mutic S , Li H . Practical considerations for noise power spectra estimation for clinical CT scanners. J App Clin Med Phys. 2016;17(3):392‐407. 10.1120/jacmp.v17i3.5841 PMC5690921 27167257

[10] Chen B , Christianson O , Wilson J , Samei E . Assessment of volumetric noise and resolution performance for linear and nonlinear CT reconstruction methods. Med Phys. 2014;41(7):071909. 24989387 10.1118/1.4881519

[11] Hacker MJ , Ratcliff R . A revised table of d' for M‐alternative forced choice. Perc and Psych. 1979;26(2):168‐170.

[12] Burgess AE , Wagner R , Jennings R , Barlow H . Efficiency of human visual signal discrimination. Science. 1981;214(4516):93‐94 7280685 10.1126/science.7280685

[13] Myers K , Barrett H , Borgstrom M , Patton D , Seeley G . Effect of noise correlation on detectability of disk signals in medical imaging. J Opt Soc Am A. 1985;2(10):1752‐1759. 4056949 10.1364/josaa.2.001752

[14] Vennart W . ICRU 54: Medical imaging—the assessment of image quality. Radiol. 1997;3(3):243‐244.

[15] Burgess AE , Li X , Abbey CK . Visual signal detectability with two noise components: anomalous masking effects. J Opt Soc Am A. 1997;14(9):2420‐2442. 10.1364/josaa.14.002420 9291611

[16] Eckstein MP , Bartroff JL , Abbey CK , Whiting JS , Bochud FO . Automated computer evaluation and optimization of image compression of x‐ray coronary angiograms for signal known exactly detection tasks. Opt Exp. 2003;11(5):460‐475. 10.1364/oe.11.000460 19461753

[17] Saunders RS , Samei E . Resolution and noise measurements of five CRT and LCD medical displays. Med Phys. 2006;33(2):308‐319. 16532935 10.1118/1.2150777

[18] Ishida M , Doi K , Loo L , Metz C , Lehr J . Digital image processing: effect on detectability of simulated low‐contrast radiographic patterns. Radiol. 1984;150(2):569‐575. 10.1148/radiology.150.2.6691118 6691118

[19] Burgess AE , Colborne B . Visual signal detection. IV. observer inconsistency. J Opt Soc Am A. 1988;5(4):617‐627. 3404312 10.1364/josaa.5.000617

[20] Rajendran K , Petersilka M , Henning A , et al. First clinical photon‐counting detector CT system: technical evaluation. Radiol. 2022;303(1):130‐138. 10.1148/radiol.212579 PMC8940675 34904876

[21] Bhattarai M , Bache S , Abadi S , Samei E . A systematic task‐based image quality assessment of photon‐counting and energy integrating CT as a function of reconstruction kernel and phantom size. Med Phys. 2024;51(2):1047‐1060. 37469179 10.1002/mp.16619 PMC10796834

[22] Fan M , Zhou Z , Bruesewitz M , McCollough C , Yu L . Evaluation of low‐contrast detectability of photon‐counting‐detector CT using channelized Hotelling observer and an ACR accrediation phantom. Proc SPIE Int Soc Opt Eng. 2023;12463:1246348. 37528865 10.1117/12.2655619 PMC10393061

[23] Zhou W , Michalak G , Weaver J , et al. Determination of iodine detectability in different types of multiple‐energy images for a photon‐counting detector computed tomography system. J Med Imag. 2019;6(4):043501. 10.1117/1.JMI.6.4.043501 PMC6792003 31620546

[24] Zhou W , Michalak GJ , Weaver JM , et al. A universal protocol for abdominal CT examinations performed on a photon‐counting detector CT system: a feasibility study. Inv Radiol. 2020;55(4):226‐232. 10.1097/RLI.0000000000000634 PMC7241672 32049691

[25] Solomon J , Samei E . Correlation between human detection accuracy and observer model‐based image quality metrics in computed tomography. J Med Imag. 2016;3(3):035506. 10.1117/1.JMI.3.3.035506 PMC5031789 27704032

[26] Richard S , Siewerdsen J . Comparison of model and human observer performance for detection and discrimination tasks using dual‐energy x‐ray images. Med Phys. 2008;35(11):5043‐5053. 19070238 10.1118/1.2988161 PMC2673596

[27] Gang GJ , Lee J , Stayman JW , et al. Analysis of Fourier‐domain task‐based detectability index in tomosynthesis and cone‐beam CT in relation to human observer performance. Med Phys. 2011;38(4):1754‐1768. 21626910 10.1118/1.3560428 PMC3069989

[28] Cruz‐Bastida JP , Gomez‐Cardona D , Garrett J , Szczykutowicz T , Chen G , Li K . Modified ideal observer model (MIOM) for high‐contrast and high‐spatial resolution CT imaging tasks. Med Phys. 2017;44(9):4496‐4505. 28600849 10.1002/mp.12404 PMC5603220

[29] Li K , Garrett J , Chen G . Correlation between human observer performance and model observer performance in differential phase contrast CT. Med Phys. 2013;40(11):111905. 24320438 10.1118/1.4822576

[30] Vaishnav JY , Jung WC , Popescu LM , Zenf R , Myers KJ . Objective assessment of image quality and dose reduction in CT iterative reconstruction. Med Phys. 2014;41(7):071904. 24989382 10.1118/1.4881148

[31] Verdun FR , Racine D , Ott JG , et al. Image quality in CT: from physical measurements to model observers. Physica Medica. 2015;31(8):823‐843. 26459319 10.1016/j.ejmp.2015.08.007

[32] Savoy RL , McCann JJ . Visibility of low‐spatial‐frequency sine‐wave targets: dependence on number of cycles. J Opt Soc Am A. 1975;65(3):343‐350. 10.1364/josa.65.000343 1123690

[33] Burgess AE . Statistically defined backgrounds: performance of a modified nonprewhitening matched filter model. J Opt Soc Am A. 1994;11(4):1237‐1242. 10.1364/josaa.11.001237 8189286

[34] Richard S , Siewerdsen J . Optimization of dual‐energy imaging systems using generalized NEQ and imaging task. Med Phys. 2007;34(1):127‐139. 17278498 10.1118/1.2400620

[35] Favazza C , Ferrero A , Yu L , Leng S , McMillan K , McCollough CH . Use of a channelized Hotelling observer to assess CT image quality and optimize dose reduction for iteratively reconstructed images. J Med Imag (Bellingham). 2017;4(3):031213. 10.1117/1.JMI.4.3.031213 PMC5624940 28983493

