# Supplementary material for: Optimization of Low‐Contrast Detectability in Abdominal Imaging: A Comparative Analysis of PCCT, DECT, and SECT Systems
Source: Med Phys. 2025 Mar 3;52(5):2832–44. doi: 10.1002/mp.17717 (PMC12059549; doi:10.1002/mp.17717)
Supplement: Supplementary file 1 — Supporting Information [file MP-52-2832-s002.pdf]

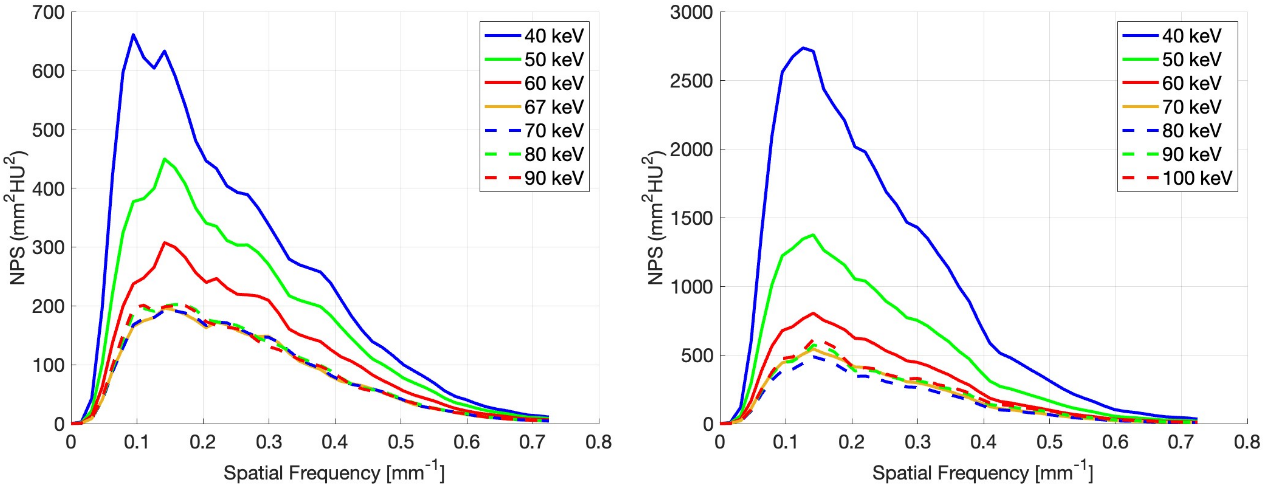

(a) PCCT.

(b) DECT.

Figure S-1: The NPS in (a) an abdominal PCCT protocol using 120 kV, and (b) an abdominal DECT protocol using 80/150Sn kV over various VMI energies are shown.
