# Supplementary figures and images for: Optimization of Low‐Contrast Detectability in Abdominal Imaging: A Comparative Analysis of PCCT, DECT, and SECT Systems
Source: Med Phys. 2025 Mar 3;52(5):2832–44. doi: 10.1002/mp.17717 (PMC12059549; doi:10.1002/mp.17717)

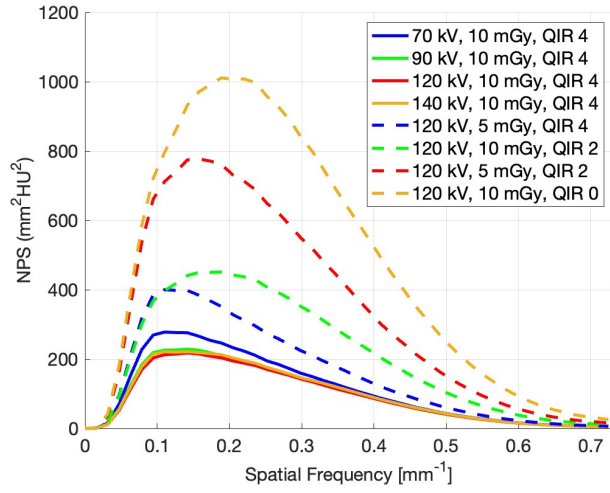

(a) NPS.

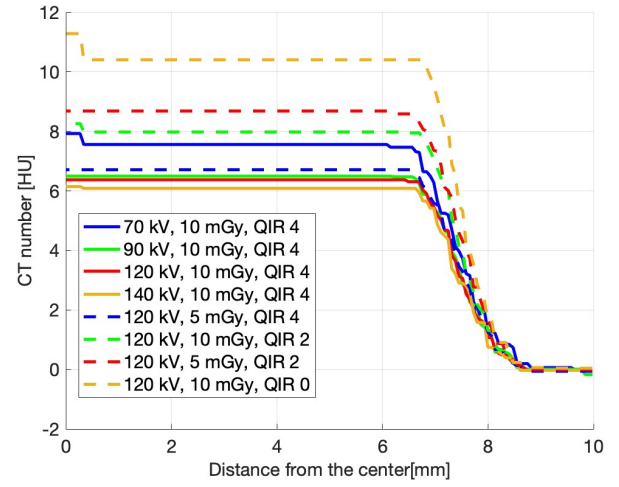

(b) ESF.

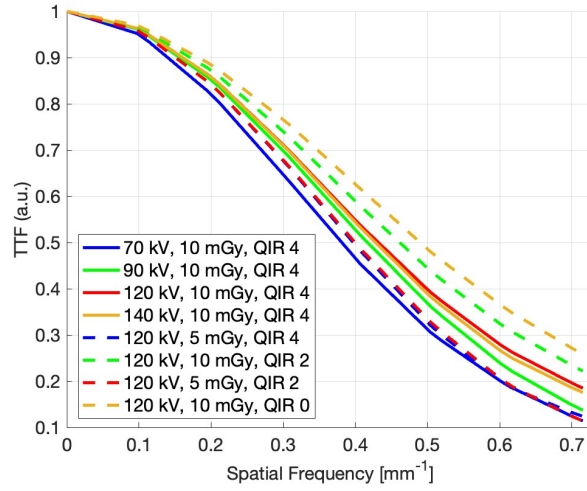

(c) TTF.

Figure S-2: The (a) NPS, (b) ESF and (c) TTF for various abdominal protocols in PCCT are shown.

Supplement: Supplementary file 2 — Supporting Information [file MP-52-2832-s003.pdf]

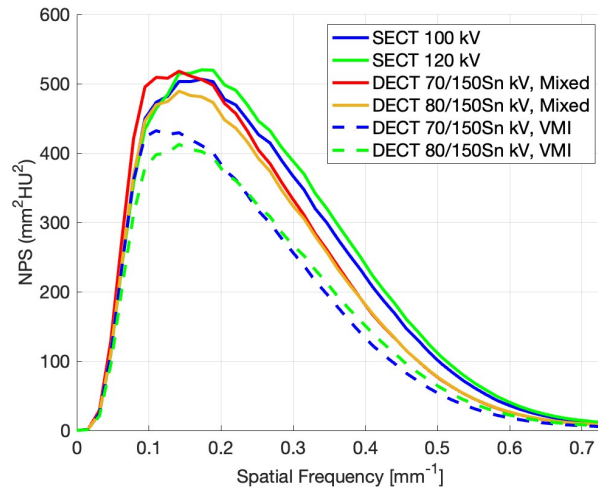

(a) NPS.

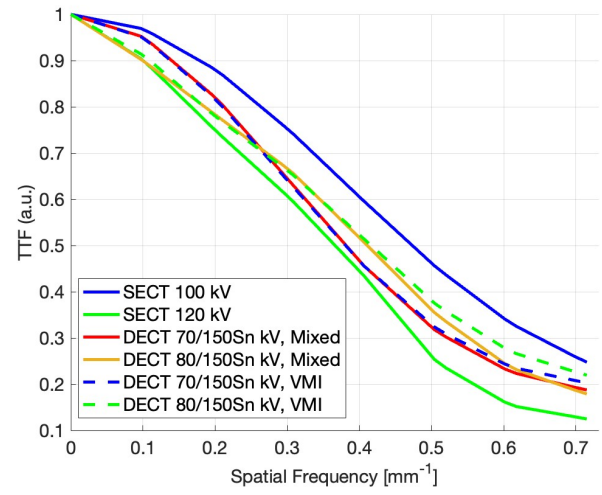

(b) TTF.

Figure S-3: The (a) NPS and (b) TTF for various abdominal protocols in DECT and SECT are shown.

Supplement: Supplementary file 3 — Supporting Information [file MP-52-2832-s001.pdf]
