# Supplementary material for: Optimization of Low‐Contrast Detectability in Abdominal Imaging: A Comparative Analysis of PCCT, DECT, and SECT Systems
Source: Med Phys. 2025 Mar 3;52(5):2832–44. doi: 10.1002/mp.17717 (PMC12059549; doi:10.1002/mp.17717)
Supplement: Supplementary file 4 — Supporting Information [file MP-52-2832-s004.pdf]

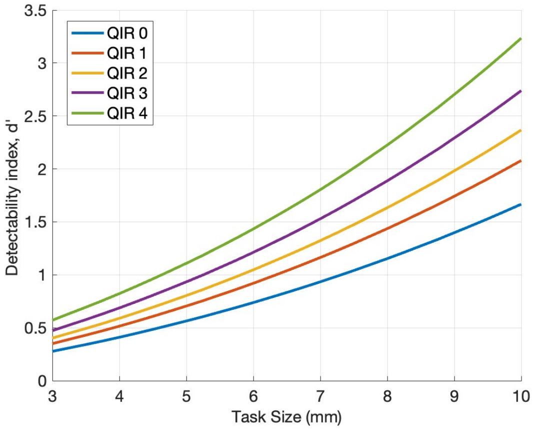

Figure S-4: For various QIR strength levels in PCCT, NPWI detectability index is shown. Tube voltage settings of 120 kV and dose settings of 10 mGy are shown.
